# Self-Reported Adherence to Medications in a Pediatric Renal Clinic: Psychological Aspects

**DOI:** 10.1371/journal.pone.0069060

**Published:** 2013-07-18

**Authors:** Tetyana L. Vasylyeva, Ruchi Singh, Christopher Sheehan, Sudha P. Chennasamudram, Anne P. Hernandez

**Affiliations:** 1 Department of Pediatrics, Texas Tech University Health Sciences Center at Amarillo, Amarillo, Texas, United States of America; 2 Department of Educational Psychology and Leadership, Texas Tech University, Lubbock, Texas, United States of America; University of Texas at Dallas, United States of America

## Abstract

**Background:**

Chronically ill children and adolescents comprise a vulnerable population that requires specific considerations in order to positively impact their treatment outcome. Pediatric renal patients can be non-compliant and also forgetful in taking their medications.

**Objective:**

The objectives of the study were to (a) assess medication adherence and (b) to identify emotionality and variables that influence non-adherence by use of “The Child & Adolescent Adherence to Medication Questionnaire” (CAAMQ), which was constructed at Texas Tech University Health Sciences Center.

**Methods:**

Pediatric renal patients from 10 to 21 years-of-age, taking three or more medications, for longer than a three-month period, were eligible to complete the CAAMQ.

**Results:**

Thirty-four patients participated in the study. Many of the respondents had problems remembering to take their medications on weekends (P = 0.021). The majority of the patients stated that they were not bothered about having to take their medications (70.6%); and that taking pills did not interfere with their daily activities (85.3%). Open-ended questions in the CAAMQ identified patients’ feelings of sadness, distress, and the importance of strong family support systems. The study participants reported that they preferred to take their medications at school, in the nurses’ office or in a place where privacy was assured. The results indicated that Prednisone was the most disliked of all of the medications. Female patients were more reactive and secretive than males regarding peers knowing about their disease and medication schedules (P<0.017).

**Conclusions:**

Non-adherence in pediatric patients is a complex and serious problem, which ultimately affects the patients’ health. Privacy and daily routine were found to impact the patients’ adherence to medications. Creative and individualized reminders for teenagers need to be developed and validated. Further studies that take into consideration developmental and motivational factors may help researchers identify modifiable psychosocial predictors that will lead to improved medication adherence.

## Introduction

Medication adherence measures the extent to which a person’s behavior corresponds with the agreed recommendations from a healthcare provider [1]. It remains one of the foremost and challenging issues for clinicians taking care of chronically ill pediatric patients. Teenagers with chronic kidney disease (CKD) and end-stage renal disease (ESRD) are in a category where adherence to the treatment plan is essential. Non-adherence to medications is one of the leading causes of acute rejection and accelerated graft failure in adolescents. The adjusted 5-year renal graft survival rates is only 57% for ages 13–21 [2]; data from the North American Renal Transplant Clinical Studies Group shows that 24% of graft failures are associated with non-adherence to medications [3].

Non-compliance with treatment among chronically ill children and adolescents with different primary diseases has been the focus of multiple studies [4]–[7]. It occurs approximately in one-third of adolescents with a chronic illness [7]. Factors associated with non-adherence include psychiatric illness, psychological factors, family issues, and health problems [7]. Poor adherence often occurs when a child reaches adolescence and is searching for greater autonomy [5]. Interventions for prevention and monitoring non-adherence include self-report, electronic monitoring devices attached to the medication container cap (medication event monitoring system, MEMS), monitoring medication usage with pill counts, prescription refill rates, and following drug trough levels [8], [9].

Improvement of adherence to medical treatment of chronically ill adolescent patients has been the objective of numerous studies [10], [11]. There is uncertainty as to which is the best approach to enhance medication adherence in this unique population [12], [13]. Dean *et al.* conducted a systematic review of the research in order to identify specific interventions aimed at improving adherence with long-term medications [10]. The study showed that educational interventions alone are insufficient in promoting compliance in children and adolescents; and that incorporating a behavioral component may increase potential efficacy [12]. Non-adherence in adolescents is not a unique phenomenon for chronically ill renal patients. Teens with asthma, diabetes, HIV, and other groups demonstrate similar behavior [10], [14], [15]. Unfortunately, there is no gold standard for measuring adherence [16]. There are numerous quality of life instruments in respect to diseases (asthma, multiple sclerosis, diabetes, *et ctr*.), but there is no unified tool to measure adherence with medications in pediatric renal disease. A deep understanding of the daily life of the chronically ill, medicated patient is essential in order to better understand their nonadherence.

The objectives of the study were to (a) assess medication adherence in pediatric renal patients and (b) to identify emotionality and variables that influence non-adherence by use of “The Child & Adolescent Adherence to Medication Questionnaire” (CAAMQ). After an extensive review of the literature, it was determined that a child and adolescent, renal, medical adherence questionnaire did not exist. In 2010 the pediatric nephrology section at Texas Tech University Health Sciences Center developed “The Child & Adolescents Adherence to Medication Questionnaire” (CAAMQ). The instrument was constructed to identify factors that can influence patients’ non-adherence to medications.

## Methods

Data was collected at Texas Tech University Health Sciences Center (TTUHSC) pediatric nephrology clinic in Amarillo, Texas. The protocol, written forms of informed consents, and assents were approved by the TTUHSC Institution Review Board for the Protection of Human Subjects. Prior to admission into the study, patients older than 18 years of age signed informed consents. In patients under 18 years of age, both signed assents and signed parental or guardian consents were obtained. Although the CAAMQ does not request the patients’ name, it does contain questions on age and diagnose; making it possible to identify the patient. All of the study documents (consents, assents, and questionnaires) were kept in a locked cabinet and data was stored in password protected computers.

The psychometric evaluation of the CAAMQ included an undeclared pre-test. The design of the questionnaire and wording was carefully formulated for the target age group. Twelve pediatric renal patients from the TTUHC clinic participated in the pre-test. One of the pre-test questions, which addressed siblings, was omitted because not all of the participants had them. The pre-test was revised for face, construct and content validity. None of the pre-test study participants reported any psychological or physical distress from their participation. All questions were in the English language and were designed for native or fluent speakers.

Patients aged 10–21 years-old and taking three or more medications, for longer than a three-month period, were eligible to participate in the study. The participants were followed in the renal clinic for one of the following diseases: CKD, ESRD or hypertension (HTN). The study took place from 06/01/2010 to 06/30/2011. Patients answered the questionnaire in the medical examination room after their regularly scheduled visit with the pediatric nephrologist. The CAAMQ consists of 19 questions: two demographic (age and gender); one medical (diagnosis); nine close-ended and seven opened-ended questions. The open-ended questions allowed the participants to freely express their opinions. Table 1 contains 15 of the questions and the remaining four open-ended questions are detailed in the Results Section.

**Table 1:** Selective questionnaire response results among 10–21 years old patients.

Questions	Answers
1. For what condition(s) are you taking medicine? (n; %)	CKD (22; 64%),ESRD (6; 18%),HTN (6; 18%)
2. How old are you? (years) (mean±SD)	15±2
3. Gender (n; %)	Male (19; 55.8% ),Female (15; 44.1%)
4. How many days per week are you supposed to be taking your medicine? (mean±SD)	6.88±0.69
5. How many days per week do you forget to take your medicine? (mean±SD)	1.04±1.73
6. Which of your medicines do you dislike the most?	Prednisone“The chunky ones”Sodium Bicarbonate
7. Does it bother you to take medicines every day? (n; %)	Yes (20; 58.8%);No (14; 41.2% )*
8. Do you feel upset about taking your medicines? (n; %)	Yes (10; 29.4% );No (24; 70.6%)*
9. Do the medicines interfere with your daily activities? (n; %)	Yes (5; 14.7% );No (29; 85.3%)*
10. Where do you most often forget to take your medicine? (n; %)	Home (12; 35.3% )*;School (1; 2.9%);Both (1; 2.9%);Never forget (20; 58.8% )
11. When do you most often forget to take your medicine? (n; %)	During Weekend (11; 32.4%)*;During Week (1; 2.9%);Both During Weekendand Week (2; 5.9% );Never (20; 58.8%)
12. Do you care about what your friends and classmates think about you needingto take your medicine? (n; %)	Yes (7; 20.6% );No (23; 67.6% )*;I don’t know (4; 11.8%)
13. Do you think an alarm would help you remember to take your medicine? (n; %)	Yes (15; 44.1% );No (14; 41.2% );I don’t know (5; 14.7%)
14. Do you think a pill box would help you rememberto take your medicine? (n; %)	Yes (19; 55.9%);No (14; 41.2% );I don’t know (1; 2.9%)
15. Do you think a better tasting medicine would help you rememberto take your medicine? (n; %)	Yes (17; 50.0% );No (10; 29.4% );I don’t know (7; 20.6%)

n = number of study participants; *P≤0.05.

Statistics: Simple statistics were used to analyze the data. In addition, a one-sample t-test between proportions was performed to determine whether respondents are more likely to prefer one alternative or another. A two-sample t-test between proportions was used to determine whether or not there was a significant difference between the male and female responses. StatPac program (Bloomington, MN) was used to perform the statistical calculations.

## Results

Thirty four patients from the pediatric renal clinic, who were prescribed more than three medications, for more than three months, took part in the study (Table 1). The average age of the population was 15±2 years, with 19 males and 15 females participating. In review of the responses by the participants (Table 1) we were able to determine that it does bother the patients to have to take medications to treat their chronic illnesses every day (P = 0.01). However, the majority of the children (P = 0.01) stated that they “do not feel upset about taking medicines” and also reported, that the medications did not interfere with their daily activities (P<0.0001).

The vast majority of teens, who admitted that they forgot to take their medicines, stated that it predominantly happened at home (12 out of 14 patients). Only 2.9% of the study population forgot to take medications at school. And again, 2.9% posited that they forgot at school and at home.

Overall, 14 patients admitted to not being adherent in taking their medications consistently, *versus* 20 who stated that they “never forgot” (P = 0.55). The majority of the study participants admitted they were less adherent on the weekends (P = 0.021).

A large number of the respondents stated that they did not care if their friends and classmates knew that they had to take medications (P = 0.002). The patients’ had mixed opinions as to what would actually help them achieve adherence: alarms (P = 0.855), pill boxes (P = 0.385) or better tasting medications (P = 0.175). Prednisone was cited as the most disliked medication followed by Sodium Bicarbonate.

The following four questions (opened-ended) allowed patients to freely express their true thoughts and emotions about having to take daily medications.

For example:

*1. How do you feel about needing to take a lot of medicine?*

Negative attitudes: “Get’s stressful”; “I feel sick, like why do I got to take them”;

“That I am not normal and very sick”; “I feel like death”;

“Like an old man” and “Mad, I don’t like it”.

Positive attitudes: “It’s my health”; “It helps me”; “It makes me feel that I am taking care of myself” and “Don’t like it, but it helps me”.

*2. What might help you feel more comfortable about taking your medicines at school?*

“Going to the school nurse”; “Taking it in the nurse’s office”; “It is a pain to have to carry pills around”; “Being alone or knowing that everybody understands” and “Privacy”.

*3. What might help you feel more comfortable about taking your medicines at home?*

“My family not pressuring me”; “Knowing that my family understands what I am going through”; “Taking fewer pills” and “Knowing that it is temporary”.

*4. Let us know of other ideas that would help remind you to take your medicine?*

“An alarm watch”; “Text messaging alarm”; “Shots – because no one has to see it”;

“A big magnet on the fridge”; “Having medication on the nightstand”;

“Make a habit to take with meals at mealtime”; “Hang it from your doorway, so you run into it and it will remind you” and “Better tasting and smaller pills”.

Gender differences were noted in adherence to medications (Table 2). The study showed that girls care more about what their friends’ think in regards to their illness and the necessity to have to take daily medications. Whereas more boys (P = 0.05) stated that they were upset that they had to take medications.

**Table 2:** Gender difference in adherence to medical treatment.

Gander	Frequency of forgettingto take medication(Days per week)	Care about whatfriends think (n; %)	Botheration abouttaking medications(n; %)	Upset for having totake medications(n; %)	Influence on dailyactivity (n; %)
Boys	1.29±1.76	Yes: 1; 5%	Yes: 11; 58%	Yes: 3; 16%	Yes: 2;11%
n-19		No: 14; 74%	No: 8; 42%	No: 16; 84%	No: 17; 89%
		Undecided: 4; 21%			
Girls	0.73±1.71	Yes: 6; 40%*	Yes: 9; 60%	Yes: 7; 47%*	Yes: 3; 20%
n-15		No: 9; 60%	No: 6; 40%	No: 8; 53%*	No: 12; 80%
		Undecided: 0			

n = number of study participants; *P≤0.05.

## Discussion

Non-adherence to a patient’s treatment plan may be influenced by developmental growth during the adolescent period. Interventions that may have been appropriate during early childhood may become ineffective in late childhood and adolescence. Non-adherence may reflect the medical team’s lack of attention to developmental changes and stages [17]. Our respondents were predominantly in the adolescent age range. This study identified psychological factors that impeded medication adherence in adolescent patients with renal diseases.

In order to answer specific research questions it was necessary to develop the CAAMQ. This questionnaire was formulated for use with chronically ill children and adolescents suffering from renal diseases. Although it was designed for use in a pediatric nephrology clinic, the tool might be applicable for use with other chronic pediatric illnesses. Further studies, with larger population sizes, are needed to fully examine the potential use of the CAAMQ in clinical settings, and in order to identify predictive variables that impede a patient’s adherence with their prescribed medications.

Unfortunately, there is a paucity of empirical studies investigating adolescent patients’ viewpoints in relation to their renal dysfunctions and their inability to adhere to their medical treatment plan. The patients’ loss of good health can negatively impact their growth and development at a very critical stage of their lifespan. Being coerced to take daily medication can adversely affect their sense of self-esteem and self-concept. A significant number of the study participants admitted that they were bothered by having to take medications every day.

A recent study, conducted on patients with rheumatic disease, showed that adherence is poor in children and adolescents, who take more than three medications [18]. In our study, all of the populations were taking more than three drugs. The majority of the respondents in the study disliked taking Prednisone followed by Sodium Bicarbonate. Non-adherence to Prednisone was found in multiple other studies [19], [20], [21]. Zerah L. *et al* [15] showed that patients have “concerns” about adverse events from taking this pharmaceutical and also the fear of drug dependency. He also posited that these patients may need both education and reassurance.

Zedler *et al*, showed that patient forgetfulness is common in unintentional non-adherence [22]. Interestingly, most of our patients had more problems remembering to take their medications on weekends, when their days are less structured, and entail more exciting activities. Vervloet *et al.* studied the efficacy of using electronics and testing reminder systems such as the Real Time Medication Monitoring with SMS, which was utilized when the patients forgot to take their medications [23].

During adolescence, the individual is focused on physical appearance, peer relationships, dating and developing a sense of identity. It is a time filled with dream and aspirations for success in the future. Adding a chronic illness during this critical period in development can result in patients with poor self-image, depression and anger; which can negatively impact the severity of their disease [24]. Research has shown that mood disorders are common in children and adolescents suffering from chronic diseases [25]. As kidney function deteriorates, the impact on the patient’s health becomes more pronounced, resulting in an increase in medications and doctors’ visits [26]. It has been shown that a debilitating disease combined with the social and psychological responses to a chronic illness can cause or exacerbate depressive symptoms [27]. Depression can negatively influence intentional non-adherence [28]. MacDonell’s recent study on HIV in youth, observed that forgetfulness, not feeling like taking medication and not wanting to be reminded were the most common barriers [29].

Most of the participants in our study said that they were not upset that they had to take medication, and that the medications did not interfere with their daily activities. The study clearly revealed that demographics and privacy, can affect adherence for pediatric renal patients. The open-ended questions in the CAAMQ provided the patients the opportunity to express their true feelings.

The concept of supporting adherence necessitates that the practitioner stays in dialogue with the patient and be available to provide time, privacy and patience; so that the adolescent may overcome their inhibitions and accept the recommended treatment [30]. It has been observed in the clinic that young patients with supportive families are more consistent in following their treatment plans. Comments like “knowing that my family understands what I am going through might help me” supports the hypothesis that chronically ill teenage patients need family understanding and support.

Attention needs to be given to the development of cell phone applications that would remind chronically ill patients to take their medication. Pediatric nephrologists need to educate their patients and families about the specific nephrological diseases that they are suffering from, the importance of following their treatment plan, and the logical consequences of non-adherence. To improve medication adherence, joint efforts between researchers, nephrologists, primary care physicians, school nurses, and parents needs to be established.

### Conclusion

Non-adherence by pediatric renal patients to their treatment plan is a complex problem that can profoundly affect the patients’ disease outcome. Privacy and daily routine were found to impact child and adolescents medication adherence. Creative and individualized reminder systems need to be developed and validated. Further studies in developmental and motivational psychology might help researchers identify factors that will lead to improved pediatric medication adherence.
